# Literature review on generational inheritance model and applications for Chinese family businesses

**DOI:** 10.3389/fpsyg.2022.885752

**Published:** 2022-11-18

**Authors:** Shuqi Li

**Affiliations:** ^1^School of Finance and Economics, Jiangsu University, Zhenjiang, China; ^2^Dongfang College, Zhejiang University of Finance and Economics, Zhejiang, China

**Keywords:** family business, inheritance model, inheritance intention, generational inheritance, inheritance stage

## Abstract

At present, Chinese family businesses have ushered in the peak period of generational inheritance, so the issue of family business inheritance has become more and more important. The generational inheritance of family businesses is not something that can be accomplished in a short period of time, but requires a long process. There are many influencing factors, and how to choose an effective inheritance model for family businesses has always been the focus of academic circles and practitioners. At present, there is no unified conclusion about the choice of family business generational inheritance model and the factors that affect the model choice. By sorting out a large number of domestic and foreign literature, this paper summarizes the predecessors’ perspectives on the inheritance model, discusses various driving factors that may affect the current inheritance model. By further analyzes the current influencing factors on the inheritance model, this paper discusses that inheritance intention of successor may be a research perspective that has not been valued, thus proposing an expandable direction in the field of family business inheritance research.

## Introduction

Family firms are the most common type of firms in the world. Gersick (1998) and others mentioned in “The Proliferation of Family Businesses” that the most conservative estimates believe that family-owned or operated businesses account for 65–80% of the world’s businesses. The “Report on the Development of Chinese Family Businesses” issued by the China Private Economy Research Association Family Business Research Group (2010) of the China Private (Private) Economy Research Association stated that among the 3,847 private enterprises in the sample surveyed, the proportion of family businesses reached 85.4%, and it accounts for more than 80% of the most industry, which can be regarded as an important force in private enterprises.

But at the same time, Chinese family businesses are also facing with the test of business continuity. The vast majority of Chinese existing family businesses were born after reform and opening-up in 1978 and are concentrated in Jiangsu, Guangdong, Zhejiang, Shandong, Shanghai and Hubei provinces. The average age of more than half of family business owner is over 55, thus Chinese family businesses are now faced with one of the largest family transitions in history. At the same time According to the survey, 44.3% of family business owners have not clearly planned the inheritance object and inheritance method of their family business (Liu, 2012).

Therefore, generational inheritance has always been a hot topic in the field of family business research. However, around the world, the rate of family businesses that can be successfully passed from one generation to the second generation is about 30%, while only 10% of family businesses can successfully pass on from the second generation to the third generation. The reason for the failure of many enterprise inheritance is that the selection of inheritance mode is not reasonable enough.

For Chinese family business, most are small and medium-sized enterprises, with 75.2% of the owners’ equity below 10 million yuan. They are mainly engaged in manufacturing, wholesale and retail. In the process of development, relatively small family enterprises have more disadvantages such as conservatism and nepotism. Family owners’ coddling of their children will lead to the “rich second generation” becoming the “negative second generation,” thus leaving no successors in the family business. At the same time, due to the significant differences between the growth background and educational background of the second generation of family enterprises and the first generation, as well as the current economic background in China, the second generation tends to prefer the financial area when choosing the direction of employment, but does not have a strong willingness to inherit the family business, leading to the potential cessation of family business which might do not have suitable successor.

It can be seen that the effective selection of the inheritance model has certain practical significance not only for the economic development of the enterprise but also for the realization of the non-economic goals of the family itself. At the same time, as an important part of the national economy, generational inheritance of family businesses are an important way to ensure the sustainable development of our country’s economy.

## Definition of family business and generational inheritance of family business

### Definition of family business

Family business is a unique enterprise form, which has certain characteristics of the enterprise and its inherent family characteristics at the same time. Alfred (1977), a well-known American business historian, first defined the family business, and believed that the traditional individual business, that is, the two-in-one business, is a family business. A family business is one where the entrepreneur and their closest partners (and families) have always held the majority of the equity. They maintain close personal relationships with managers and retain major decision-making powers for senior management, especially regarding financial policies, resource allocation, and selection of senior executives. In addition, academic circles have also tried to define family business more accurately from various perspectives, including the perspective of family business shareholding, the actual operation and management of family business, and the combination of the two.

#### From the aspect of ownership

For the identification of family business, some scholars believe that it can be defined by assessing whether the family can fully control the business from the perspective of ownership. Donckels and Frohlich (1991) considered a family business as a type of business that family member holding 60% or more of the company’s equity. Gersick and Davis (1997) also proceeded from ownership and believed that no matter whether the enterprise is named after the family or the relatives are in the high-level leadership of the enterprise, it cannot be determined that a certain enterprise is a family enterprise. The only factor of deciding it is a family business is family ownership.

#### From the aspect of management rights

Some scholars believe that the ownership of a family business to the enterprise cannot represent the unique attributes of its family characteristic, but should be defined from the perspective of management rights. Donnelley (1988) proposed that a family business refers to the fact that at least two generations of the same family are involved in the operation and management of the company, and because of the connection between the two generations, the company’s policies and the interests of the family influence each other. In addition to considering corporate policies, some scholars have proposed that companies whose main operations and succession decisions are influenced by family members who hold management positions or directorships in the company can be called family businesses (Handler, 1989). In addition, if there is equity owned by the public, the family needs to control the operation of the enterprise so that the enterprise can be defined as a family enterprise (Pat, 1982). Sun (1995) directly combined the definition of a family business with the management right of an enterprise, arguing that when a family or several families with close alliances directly or indirectly hold the management right of an enterprise, the enterprise is a family enterprise.

#### From the aspect of combination of ownership and management rights

As the research direction of the generational inheritance of family business continues to spread, the definition of family business in academia is constantly being supplemented. Therefore, scholars’ definition of family business is not satisfied with a single dimension, but more fully describes and defines the definition of family business by combining ownership and management rights. When a family owns all or part of an enterprise and directly or indirectly holds the right to manage the enterprise, the enterprise is a family enterprise (Pan, 1998). Ye (1992) defined a family business as a family business with the following three conditions: (1) The shareholding ratio of the family is greater than the critical shareholding ratio; (2) Family members or relatives within the second-degree relatives serve as chairman or general manager; (3) Family members or relatives within the third degree of relatives hold more than half of the company’s board seats. Similarly, Bennedsen et al. (2015) also suggested that family businesses should have the following characteristics: at least two members of the same family play a role in corporate governance, family equity dominates the business, family members enjoy absolute control over the business, and companies prefer to choose the same for generational inheritance and alternation within the family.

It can be seen that the definition of family business has gradually become complete by starting from different dimensions, but a representative unified opinion has not yet been formed. Therefore, for the definition of family business in China, on the basis of considering the characteristics of family culture, the definition of family business is taken from the perspective of combining ownership and management rights, and at the same time, the characteristics of family business such as corporate culture may be considered. More generally recognized and accepted.

### Definition of generational inheritance of family business

The characteristics of family businesses include both ownership and management rights related to the enterprise, so the generational inheritance of family businesses can also be understood as the process of the owner of the business passing the ownership and management rights to the successors. The generational inheritance of family businesses is a long-term socialization process. Therefore, in the process of generational inheritance, changes in the business environment, evolution of industry development, adjustment of strategic goals, changes in family needs and goals may all lead to changes in the choice of successors (Bird et al., 2002). There are also various definitions for the description of inheritance, depending on the specific content of the inheritance. Stavrou (1998) believes that generational inheritance is mainly the transferring of corporate leadership positions; Ambrose (1983) believes that generational inheritance is the transfer process of corporate ownership from one generation to the next generation; Murray (2003) more comprehensively believed that the inheritance is not only the management right of the enterprise, but also the ownership of the enterprise, and the transfer of both can be considered as generational inheritance. In addition to the ownership or control in the traditional sense, some scholars have supplemented it from other heritage content. Dyck et al. (2002) described it as the transfer of property rights and execution rights, while Bao and Yongbin (2020) believed that inheritance is not only explicit control and ownership, but also implicit authority transmission between generations.

If a family business wants to be more competitive under the fierce market competition and the survival law of the survival of the fittest, it must be able to successfully achieve generational inheritance. According to the different inheritance content that various definitions of generational inheritance focus on, it can be seen that the study of the generational inheritance model of family businesses should firstly clarify “what to pass on,” and then analyze “how to pass it on.” The transition from the transfer of tangible resources to intangible resources is a topic of common concern in the generational inheritance of family businesses.

## Theoretical basis of family business inheritance

The research on family business in western countries is earlier, Gersick and Davis (1997) put forward the well-known three-ring model and three-level development model, which has always been the classic model of family theory research.

### Three-ring model

This model thinks of a family business as a three-ring system consisting of three independent but intersecting subsystems: business, ownership, and family. Any individual in the family business can be placed in one of the seven areas formed by the intersection of these three subsystems.

All owners are in the top ring, all family members are in the bottom left ring, and all businesses are in the bottom right ring. Each individual that is a member of the family business system has a definite place in this model. By examining the individual’s position in the three rings, it is possible to clarify the boundaries of rights, responsibilities and authorities of individual opinions, which can help the family business to split up the internal interactions.

However, this model only reflects the static state of the family business at some specific moments, and does not consider the dynamic development and constant change of the family business.

### Three-level development model

Since the three-ring model does not take into account the positional migration of individual family businesses over time, Gersick et al. (1999) further proposed a three-level development model for family businesses. This model reveals the life cycle of family members in the family business, the relationship between the life cycle of the business and the change of ownership of the family business.

For the inheritance process, scholars at home and abroad have also proposed relatively rich theoretical models, the more influential ones include:

### Cognitive classification path analysis model

By combining the inheritance process with the cognitive level and cognitive evaluation of entrepreneurs and successors, Matthews et al. (1999) on the basis of the seven-stage succession model, summarized succession as the successor entering the enterprise as a full-time employee and father-son model leadership. There are two stages of inheritance. Further analysis found that: when the leaders of the parent generation feel that they need to develop new hobbies or the possibility of death is going to increase, they often no longer block the formulation of leadership handover plans; the leaders of the parent generation are interested in the leadership ability of their children, which helps to expand the successors of their children. Whether the children actively strive for the status of successor depends on their self-assessment of their ability to adapt to the working environment; if the children have a positive evaluation of the leadership ability of their parents, they will adopt a negative attitude towards seeking succession, and vice versa. If the leadership ability of the parents is negatively evaluated, then they will actively seek succession.

### Inheritance cycle model

Through a longitudinal comparative study of multiple cases, Murray (2003) further increased the cyclical impact on the six-stage model, and developed a more detailed model for the generational inheritance process of family businesses in terms of time. He believes that the entire process needs to continue 3–8 years.

Generational inheritance is one of the primary concerns in the field of family business research, and the theoretical model is constantly being updated. From the initial three-ring model and three-level model that only focused on individual positioning and individual positioning migration, to the stage model that gradually focused on the process view, the dynamic process of generational inheritance has attracted more and more attention from scholars, and is also considered to be the realization of the family. The primary condition for the smooth succession of an enterprise (Dou and Jia, 2007).

### Additional approaches

Besides the famous models that have been mentioned above, other detailed models which have focused on different priorities also play a key role in the following research. The first one is the **life stage model**. Davis and Tagiuri (1989) studied the relationship between fathers and sons in the succession process and found that the best time for power transfer is after the age of 50 for parents, and when their children are 27 to 33 years old. For most family businesses, there are often contradictions in the inheritance time node. The second one is **three stage model.** Handler (1989) proposed a three-stage model based on the model of life stages, and summarized the process of inheritance into three stages: personal development stage, enterprise development stage and leadership inheritance stage. The third one is **seven stage succession model.** Longenecker and Schoen (1991) proposed a seven-stage succession model, which described the overall process of generational inheritance in a more detailed manner., early succession and formal succession. The fourth one is **three-level generational inheritance model.** Stavrou (1998) from the perspective of intergenerational level progression, through the method of combining literature analysis and empirical analysis, the family business inheritance process is divided into three stages: before children enter the enterprise, entering the enterprise and formal succession. The last one is **six stage model.** Gersick et al. (1999) further divided the inheritance process of family business ownership into six stages: pressure accumulation, triggering, disengagement, exploration of options, selection, and implementation of new structures on the basis of the three-level development model.

The core of all the mainstream intergenerational inheritance models that have been mentioned above lies in the characteristics of periodic transmission between generations, focusing on the process and stages. However, as the inheritance laws differ significantly in the world, the above models are more suitale for the mode selection of Western countries for the inheritance of offspring. Domestic intergenerational inheritance research focuses more on the selection of the process. Therefore, no more groundbreaking theory has been put forward in China and most scholars in China are still trying to use the foreign theories or the adjust the theories for Chinese family business. And also from the perspective of the relatively mature inheritance theories in foreign countries, most of them focus on the early cultivation of successors, the formulation of inheritance plans, and the selection of professional professional managers when there is a lack of suitable successors. The whole inheritance process cycle is long and the plan is solid, such as life cycle model and three-level development model. However in China, most family business owners lack the planning of inheritance and potentially have the stronger desire to keep power for themselves. Thus they might have not set up a special successor training program, and only temporarily replace business owners when stuck with problems such as age and health issue. Thus under the background that Chinese family enterprises pay more attention to family culture, how to effectively realize intergenerational inheritance is in urgent need of a set of theories that better fit the reality of Chinese family enterprises.

## The mode of generational inheritance of family business

After accumulating a certain amount of wealth, a large number of family businesses have all need to consider the issue of successors, but how to choose the successor and how to successfully realize the business handover have become the problems faced by the family business. Therefore, a large number of scholars have conducted a full analysis of the type of successor to choose. Due to the relatively early research on family business in the West, the model of generational inheritance is relatively full. The traditional inheritance models are mainly divided into two types: internal inheritance and external professional manager market (Boyd, 2012). For the inheritance model, when there are more than one internal inheritor, the internal inheritors can be further subdivided into different categories. For example, He et al. (2013) proposed that internal inheritance includes primogeniture inheritance, merit-based inheritance, and inheritance by various sons. There is also a view that when the number of internal heirs is insufficient, the willingness to inherit is not strong, or the succession ability is insufficient, a new division of the inheritance model can be made, which can be divided into family members, endogenous training and external managers (Liao et al., 2021). At this time, endogenously cultivated heirs are different from directly hired managers. They may have a better understanding of the enterprise, and at the same time, the possibility of generating agency costs will be reduced. In addition, in this context, it is also possible to consider using veterans as heirs (Zhong, 2009).

If the inheritance process is considered as a long-term stage, the division of inheritance patterns is more refined. Chen (2018) proposed that family business succession models can be divided into five categories, including the joint operation of the business by heirs and professional managers; handing over the business to professional managers; the complete transfer of the original business, and the children starting a new business; “pocket” model that is to divide the property into three parts: enterprise, cash and foundation, which are inherited, respectively.

Although it is generally believed that there are different modes of inheritance between internal inheritance and external professional manager inheritance, combined with the cultural background of our country and the first goal of the development of most family businesses is enterprise “safety rather than economic goals,” many scholars believe that family businesses in China are more suitable for adopting the method of internal inheritance, that is, the model of “children inheriting the father’s business” in the traditional sense.

Due to factors such as the low level of trust in Chinese society and objective factors such as the imperfection of the Chinese professional manager market, Chinese family businesses are almost always “inheriting the father’s business” in the selection of successors. However, some scholars suggest that professional managers can also be candidates for corporate successors. When family members are not qualified to complete various tasks of the company, founders must consider that professional managers and social investors are qualified inheritors of the company (Jin et al., 2020). Or by adopting some modern corporate governance models to help family businesses achieve the inheritance of management rights and ownership that cannot be accomplished by family members (Yang, 2015), that is, to further integrate multiple models and broaden the boundaries.

Thus the unplanned internal inheritance might not adapt to the rapid development of enterprises. First of all, when the business owner wants to pass on the business to his children, the power pattern and ownership distribution will change greatly, and there will be fierce conflicts between the heirs and the existing senior management of the business, and the heirs are often unable to hold substantial power. At the same time, as the long-term support of the enterprise, the owners’ weak willingness to inherit may further intensify the contradiction between the second generation as successors and the existing management, who might consist of the relative of the owners. These relatives play a major role as a shareholder will tend to discourage the second generation from inheriting the family business to avoid weakening their power. And also, as successors, the education and environment of the second generation are significantly different from those of the first generation. Due to different business concepts and strategic thoughts from the first generation, the second generation of family enterprises in China does not have a strong willingness to inherit. At the same time, due to the influence of China’s own employment environment and the rapid development of the economic background, many of the second generation choose the high-yield and high-risk financial industry as the direction of employment, while some of the second generation choose to join state-owned enterprises and other enterprises with lower risk as their first choice of employment. In the early 1970s, in order to solve the important problems of China’s large population base, rapid growth and low general quality, the government formulated a family planning policy with “late marriage, late childbearing, fewer children and healthy birth” as the main content and purpose, and subsequently made it a basic state policy of China. This policy has further affected a large number of family businesses. When there is only one successor in the enterprise, and the successor does not have strong inheritance intention or the ability to inherit the enterprise, the choice of the intergenerational inheritance mode of the family enterprise will be further seriously affected.

## Influencing factors of family business inheritance model

The choice of family business generational inheritance model is affected by many factors, from macro-level economic factors, cultural factors to micro-level corporate individual factors.

From the overall background level, due to the influence of traditional Confucianism in China, the fathers of business owners are often more inclined to choose “the sons inherit the father’s business,” even if the sons are not suitable for succession. At the same time, due to the unique pattern of differential order in China, there are differences in the degree of trust between people, which leads to strong relationships and weak organization in family businesses. In addition to cultural and economic factors, the immaturity of Chinese current professional manager market itself has also become an objective factor limiting the external succession of family businesses.

From a company-level analysis, Belmich and Brown (1972) believes that generally larger family businesses have a larger talent pool and have the ability to continuously develop and cultivate the company’s internal personnel, so large companies are less likely to choose from outside. Smaller companies may not have a suitable internal candidate and may be forced to recruit from outside. However, some scholars have proposed that, considering the company’s policy governance or company innovation, it is a more appropriate choice to choose the inheritance model of external inheritance. Wiklund et al. (2013) proposed that external inheritance is not bound by old policies and implicit contracts of enterprises, so that it can enrich the new perspectives that enterprises need, generate creative ideas and act more decisively. Minichilli and Nordqvist (2014) proposed that external inheritance can overcome the emotional pressure faced by strategic shifts such as corporate values and systems.

In addition to analyzing the influence of various external factors on the inheritance model, more and more scholars have begun to investigate the family business from the individual level, trying to find the influencing factors from within the enterprise. From the perspective of the incumbent, the generational inheritance process of family businesses is mostly carried out under the control of senior managers, especially the incumbent business owners. They decide who inherits the management rights of the enterprise, when the inheritance process starts, and how to implement the inheritance process (Beckhard, 1983; Goldberg and Wooldbridge, 1993; Lansberg, 1999). Motivated to continue to enjoy power, family business owners often lack long-term planning for inheritance, but only distribute wealth to their offspring and do not want to decentralize corporate governance. From the perspective of successors, Royer et al. (2008) believes that successors with professional knowledge are more likely to become the objects of inheritance. Sardeshmukh and Corbett (2011) believes that the successor’s self-confidence, education and work experience, innovation ability, and human capital awareness all have a positive effect on the success of the successor. Some scholars believe that the founder’s inheritance willingness, the successor’s succession ability, and the quality of the relationship between the two play an important role in the smooth implementation of inheritance due to the influence on the interaction between the incumbent and the successor. At the same time, if the values of the two generations are too different and their opinions on management are not unified, it is very likely to hinder inheritance activities and thus hinder the overall development of the enterprise.

In addition, although China had been made draft the inheritance tax law of the People’s Republic of China as early as in 2004,China has not started to make this tax in force, so for property inheritance, and wealth is not divided into mandatory provisions of law, which will further lead to the wealth of Chinese family business owners lack of long-term planning. Thus there is also a lack of long-term planning for the succession of the family business. However, in western countries, due to the existence of inheritance tax, the division of family wealth has a legal basis, so most family business owners will carry out the distribution of family wealth in advance, including the planning of the inheritance object of the family business.

Furthermore, more and more scholars have begun to further refine the inheritance model, and take how the successor achieves smooth inheritance as the further research object of the inheritance model, such as establishing authority and legitimacy through second-generation entrepreneurship or hybrid entrepreneurship to achieve smooth success inherited. In the new subdivided inheritance model, what factors will have an impact on it is also the research direction of family business research.

## Prospects for future research directions

The research on the generational inheritance of family businesses has appeared abroad since then. Scholars at home and abroad have continued to improve the research content of family businesses. From the definition of family business, the definition of family business inheritance, the exploration of inheritance mode, to the research on various subjective and objective factors that affect inheritance mode, from different perspectives, using different methods to conduct in-depth research. Generally speaking, foreign research on generational inheritance is relatively sound, and the perspectives are also richer. However, Chinese family business began to develop after the reform and opening up, with a relatively short development process and relatively short attention time. As family businesses in China are now facing with the problem of generational inheritance, the research on generational inheritance of family businesses has been enriched. However, the current research mainly focuses on the comparison and selection of external inheritance and internal inheritance. The weight analysis and integrated analysis of various influencing factors are not sufficient, especially the research perspective of successors is not enough. Therefore, this paper believes that there is still some research space for this perspective for future research. Through the research on the generational inheritance environment and the inheritance mechanism model, we can further explore the changing characteristics and laws of the dynamic generational inheritance model of family businesses, so as to explore the family business inheritance model and management system with Chinese characteristics, and realize the sustainable family business development.

## Author contributions

SQL contributed to the conception and design of the study, wrote the manuscript and contributed to manuscript revision, read, and approved the submitted version.

## Conflict of interest

The author declares that the research was conducted in the absence of any commercial or financial relationships that could be construed as a potential conflict of interest.

## Publisher’s note

All claims expressed in this article are solely those of the authors and do not necessarily represent those of their affiliated organizations, or those of the publisher, the editors and the reviewers. Any product that may be evaluated in this article, or claim that may be made by its manufacturer, is not guaranteed or endorsed by the publisher.
